# The effect of ureteral stenting on the risk of metachronous upper tract urothelial carcinoma in patients with bladder tumors involving the ureteral orifice

**DOI:** 10.1007/s00345-026-06839-7

**Published:** 2026-10-05

**Authors:** Nikolaos Pyrgidis, Patrick Engelmann, Daniel Puhr-Westerheide, Benedikt Ebner, Marc Kidess, Marie Semmler, Patrick Keller, Christian Stief, Gerald Bastian Schulz, Yannic Volz

**Affiliations:** 1 Department of Urology, LMU University Hospital, Marchioninistraße 15, 81377 Munich, Germany; 2 Department of Radiology, University Hospital, LMU Munich, Munich, Germany

**Keywords:** Urothelial cell carcinoma, TURBT, Ureteral stent, Hydronephrosis, Upper tract urothelial cell carcinoma

## Abstract

**Background:**

Ureteral stenting in patients with bladder cancer involving the ureteral orifice may increase the rate of metachronous upper tract urothelial carcinoma (UTUC). Nevertheless, evidence on the matter is scarce and often contradictory.

**Materials and methods:**

We performed an observational study comparing ureteral stenting versus no stenting with respect to the risk of developing metachronous UTUC in patients who underwent transurethral resection of bladder tumors (TURBT) involving the ureteral orifice between January 2015 and December 2024. We performed time-to-event analyses to estimate hazard ratios (HRs) along with their corresponding 95% confidence intervals (CIs).

**Results:**

We included 151 patients with a median age of 73 years. 37 (25%) underwent ureteral stenting during TURBT. Patients undergoing stenting had higher BMI (*p* = 0.014), more aggressive tumors (*p* = 0.003), underwent prior intravesical therapy more often (*p* = 0.008), higher preoperative creatinine values (*p* = 0.007), and longer hospitalization duration (*p* = 0.002) compared to those who did not undergo stenting. After a median follow-up of 25 months, 8 patients developed metachronous UTUC (2 in the stenting group and 6 in the non-stenting group). Ureteral stenting was not associated with statistically significantly higher rates of metachronous UTUC (HR: 1.2, 95%CI: 0.31–5.6, *p* > 0.9). During follow-up, 49 (33%) patients developed recurrence in the bladder, 43 (28%) underwent cystectomy, and 24 (16%) died. The HRs did not significantly differ between the two groups.

**Conclusions:**

The present study did not demonstrate an increased risk of metachronous UTUC after stenting during TURBT with urothelial cancer involving the ureteral orifice. Moreover, stenting was not statistically significantly associated with increased recurrences in the urinary bladder, higher cystectomy rates, or worse mortality.

**Supplementary Information:**

The online version contains supplementary material available at https://doi.org/10.1007/s00345-026-06839-7.

## Introduction

Upper tract urothelial carcinoma (UTUC) accounts for approximately 10% of all renal and 5% of all urothelial tumors, with an estimated incidence of two cases per 100,000 individuals annually in the United States [1]. UTUC and bladder cancer share similar molecular and pathogenetic characteristics, which explains why a considerable number of UTUC patients have a prior history of bladder cancer or are diagnosed with both diseases simultaneously [2]. This overlap is thought to result from an epigenetic “field effect” that predisposes the entire urothelium to malignant transformation [3]. Accordingly, a small proportion of patients initially diagnosed with bladder cancer will later develop synchronous or metachronous UTUC [4].

In patients with bladder cancer involving the ureteral orifice, drainage of the upper urinary tract, through retrograde/antegrade ureteral stenting or percutaneous nephrostomy, may be necessary to relieve obstruction during transurethral resection of bladder tumors (TURBT) [5]. Some experts advise against routine stenting following TURBT involving the ureteral orifice, as it may be associated with a higher risk of metachronous UTUC [6] and suggest placing a percutaneous nephrostomy in cases of persistent postoperative hydronephrosis [7]. However, evidence on this issue remains inconsistent [8].In line with that, other investigators report no significant association between ureteral stenting during TURBT and the development of metachronous UTUC, and therefore continue to favor retrograde stent placement when resection involves the ureteral orifice [9–11]. Consequently, the decision to insert a ureteral stent is often left to the individual surgeon’s discretion [12].

Adding to this uncertainty, current guidelines, based on a meta-analysis of solely retrospective studies [13], recommend avoiding ureteral stenting during TURBT, as prospective studies addressing this question are still lacking [14]. In this context, we evaluated whether ureteral stenting after resection of the ureteral orifice during TURBT represents a risk factor for the subsequent development of metachronous UTUC in patients with bladder cancer.

## Materials and methods

### Study design and selection criteria

The present observational study was performed at the Department of Urology, University Hospital, Ludwig Maximilian University of Munich, Germany. The study was conducted in accordance with the Declaration of Helsinki, was approved by the institutional ethical committee, and its findings were reported based on the STROBE statement for cohort studies [15]. We included only adult patients who underwent TURBT in our department between January 2015 and December 2024 for primary or recurrent non-metastatic urothelial bladder cancer involving the ureteral orifice. We excluded patients with prior or concomitant UTUC, as well as those with variant histology or non-bladder cancer. Subsequently, all included patients and/or their ambulatory urologists were contacted between August 2025 and October 2025 for an additional in-person or telephonic evaluation to assess their tumor progression/recurrence, as well as to evaluate whether they developed metachronous UTUC at the site of the initial resection of the ureteral orifice during TURBT.

Patients underwent a standardized schedule for upper-tract imaging according to the EAU guidelines. In particular, a CT was performed annually for up to 5 years in all high- or very high-risk tumors. Otherwise, all patients underwent regular ultrasound evaluations, and a CT was performed when UTUC was clinically suspected. Surveillance practice was similar between the stented and non-stented groups. Furthermore, patients who underwent radical cystectomy followed the corresponding EAU standardized follow-up for muscle invasive bladder cancer.

### Data collection and outcomes

The perioperative data of all included patients were collected from their medical records, and their follow-up data were obtained during the additional in-person or telephonic evaluation. Data on baseline characteristics, oncological status, perioperative findings, disease recurrence or progression, as well as laboratory values, were retrieved. The primary outcome of the present study was to assess whether ureteral stenting after resection of the ureteral orifice during TURBT represents a risk factor for the development of metachronous UTUC in patients with bladder cancer. Stenting was defined as the placement of a ureteral stent either during the initial TURBT or within 4 weeks postoperatively. Any stent placement performed later, as well as the absence of stenting, was classified as no stenting. Secondary outcomes included recurrence rates of bladder tumor, cystectomy rates, as well as overall survival. Treatment allocation (stenting or no stenting) was surgeon-dependent and non-randomized.

### Statistical analysis

Continuous variables were summarized as medians with interquartile ranges (IQRs), and categorical variables were presented as proportions. The comparisons between the two groups were performed using the two-sample t-test or the Mann-Whitney test. Kaplan–Meier curves with the log-rank test were also used to evaluate the effect of ureteral stenting on metachronous UTUC. For all time-to-event outcomes, we estimated hazard ratios (HRs) with corresponding 95% confidence intervals (CIs) using competing-risk regression. A two-sided p-value < 0.05 was considered statistically significant for all outcomes. All analyses were performed using the R statistical software.

## Results

### Baseline characteristics

From 2,980 patients undergoing TURBT in our department between January 2015 and December 2024, we included 151 patients who received resection of the ureteral orifice and fulfilled our selection criteria. Their median age was 73 years (IQR: 64–80), their median BMI was 26 kg/m^2^ (IQR: 23–28), and 112 (74%) patients were male. A total of 69 (53%) patients were smokers, 71 (47%) had hypertension, and 21 (14%) had diabetes mellitus. Thirty-seven (25%) underwent stenting during TURBT, and 114 (75%) received no stenting despite resection of the ureteral orifice. The median operative time of TURBT was 51 min (IQR: 36–68), and the median length of hospital stay was 4 days (IQR: 4–5).

Of the 37 patients undergoing stenting, 27 (73%) received ureteral stenting during TURBT for prophylactic reasons, while 10 (27%) received stenting within four weeks after surgery due to hydronephrosis with rising creatinine. The indications and the characteristics of the patients stented intraoperatively or delayed are available as Supplementary Material. Patients undergoing stenting presented higher BMI (*p* = 0.014), had more aggressive tumors (*p* = 0.003), had undergone prior intravesical therapy more often (*p* = 0.008), had higher preoperative creatinine values (*p* = 0.007), and stayed longer in the hospital (*p* = 0.002) compared to those who did not undergo stenting. The baseline characteristics of all included patients are presented in Table 1.

**Table 1:** Baseline characteristics of patients undergoing TURBT for non-metastatic urothelial bladder cancer involving the ureteral orifice

Characteristic	Overall, *n* = 151	No stenting, *n* = 114	Stenting, *n* = 37	*p*-value
Age (years)	73 (64–80)	74 (64–80)	73 (63–78)	0.6
Males	112 (74%)	86 (75%)	26 (70%)	0.7
BMI (kg/m^2^)	26 (23–28)	25 (23–28)	27 (25–31)	**0.014**
Smokers	69 (53%)	51 (52%)	18 (55%)	> 0.9
Hypertension	71 (47%)	54 (47%)	17 (46%)	> 0.9
Diabetes mellitus	21 (14%)	14 (12%)	7 (19%)	0.5
ASA score	3 (2–3)	3 (2–3)	2 (2–3)	0.6
Operative time (minutes)	51 (36–68)	49 (35–65)	62 (41–77)	0.056
First tumor diagnosis	125 (83%)	97 (85%)	28 (76%)	0.3
T Stage				**0.003**
pTa	84 (56%)	66 (58%)	18 (49%)	
pT1	31 (21%)	28 (25%)	3 (8.1%)	
pT2	36 (24%)	20 (18%)	16 (43%)	
Concomitant pTis	20 (13%)	15 (13%)	5 (14%)	> 0.9
Multifocal tumor	62 (41%)	49 (43%)	13 (35%)	0.5
Diameter of tumor (cm)	3 (2–4)	3 (2–4)	4 (2–5)	0.3
Prior intravesical therapy	14 (9.3%)	6 (5.3%)	8 (22%)	**0.008**
Preoperative creatinin (mg/dl)	1 (0.9–1.3)	1 (0.9–1.2)	1.2 (1-1.5)	**0.007**
Hospital stay (days)	4 (4–5)	4 (4–5)	5 (4–6)	**0.002**

Values presented as median with interquartile range or n (%), Bold values indicate statistical significance

*ASA* American society of anesthesiology, *BMI* body mass index

### Metachronous UTUC

**Fig. 1:**
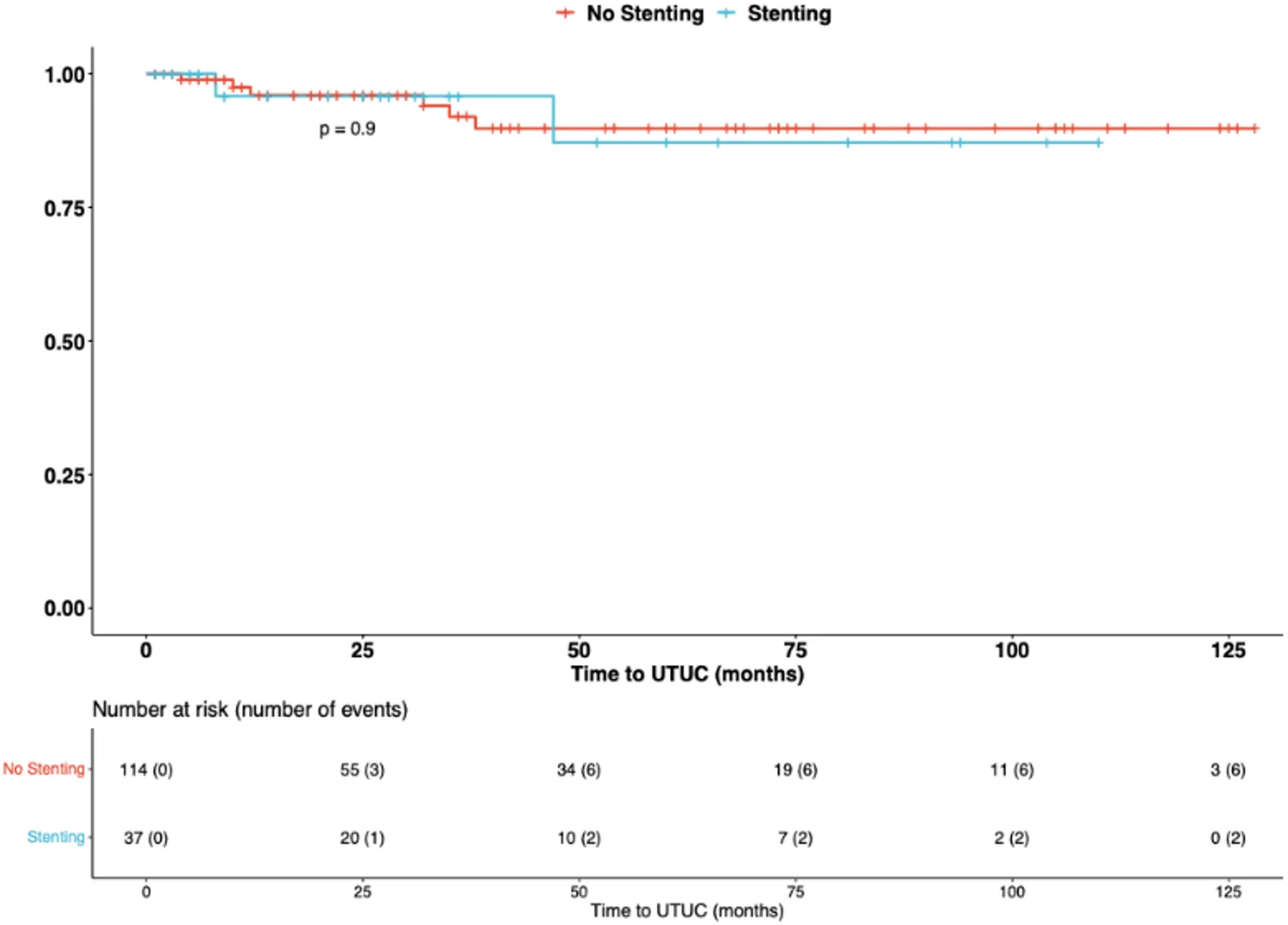
Kaplan-Meier curve for metachronous UTUC in patients undergoing TURBT for non-metastatic urothelial bladder cancer involving the ureteral orifice

At a median follow-up of 25 months (IQR: 4–60), 8 (5.3%) patients developed metachronous UTUC at the site of the initial resection of the ureteral orifice during TURBT. Of these, 6 (75%) occurred in the group without stenting during TURBT, and 2 (25%) occurred in the group with stenting during TURBT (*p* > 0.9). In the univariate Cox regression analysis, ureteral stenting was not associated with statistically significantly higher rates of metachronous UTUC (HR: 1.2, 95% CI: 0.31 to 5.6, *p* > 0.9). The corresponding Kaplan–Meier curve is depicted in Fig. 1. Moreover, no differences were observed between patients undergoing stenting during TURBT versus those who underwent stenting within 4 weeks after TURBT.

### Recurrences, cystectomy rates, and mortality

At a median follow-up of 25 months (IQR: 4–60), 49 (33%) patients developed recurrence in the urinary bladder, 43 (28%) patients underwent radical cystectomy, and 24 (16%) patients died. In the univariate Cox regression analysis, ureteral stenting was not associated with statistically significantly higher rates of recurrences in the urinary bladder (HR: 0.49, 95% CI: 0.23 to 1.05, *p* = 0.07), cystectomy rates (HR: 1.3, 95% CI: 0.65 to 2.45, *p* = 0.5), or with worse mortality (HR: 1.97, 95% CI: 0.86 to 4.51, *p* = 0.11). Major outcomes during follow-up are summarized in Table 2.

**Table 2:** Major outcomes during follow-up of patients undergoing TURBT for non-metastatic urothelial bladder cancer involving the ureteral orifice

Characteristic	Overall, *n* = 151	No stenting, *n* = 114	Stenting, *n* = 37
Metachronous UTUC	8 (5.3%)	6 (5.3%)	2 (5.4%)
High-grade UTUC	4 (50%)	4 (67%)	0 (0%)
Tumor involving the ureter	7 (88%)	6 (100%)	1 (50%)
Recurrence in the bladder	49 (33%)	41 (36%)	8 (22%)
Cystectomy	43 (28%)	31 (27%)	12 (32%)
Deaths	24 (16%)	15 (13%)	9 (24%)
Follow-up (months)	25 (4–60)	23 (4–61)	27 (2–52)

Values presented as median with interquartile range or n (%)

*UTUC* upper tract urothelial cancer

## Discussion

The findings of the present single-center cohort study suggest that stenting during TURBT in patients with urothelial cancer involving the ureteral orifice does not increase the rates of metachronous UTUC. More specifically, after a median follow-up of more than two years, patients undergoing stenting during TURBT presented similar oncologic outcomes compared to those who did not undergo stenting during TURBT. Accordingly, the two groups did not differ in terms of recurrences in the urinary bladder, cystectomy rates, or overall mortality.

There is discordance and hence variation in the clinical management of tumors involving the orifice during TURBT. The treatment plan after a TURBT involving the ureteral orifice includes either no drainage or retrograde stenting as a preventive measure to avoid ureteral orifice stricture and subsequent obstruction [16]. Most surgeons opt for no stenting, since ureteral stents may lead to metachronous UTUC through disruption of the natural anti-reflux barrier at the ureterovesical junction, allowing retrograde spread and implantation of malignant cells from the bladder into the upper urinary tract [17, 18]. Additionally, instrumentation during stent placement might contribute to tumor cell migration [19]. The decision for no stenting is further reinforced by the fact that ureteral stents may negatively affect quality of life since they are associated with lower urinary tract symptoms such as increased urinary frequency, hematuria, and dysuria [20].

Our findings are in line with previous retrospective cohort studies. Notably, Chou et al. conducted the first study to evaluate the role of ureteral stenting in patients with bladder tumors involving the ureteral orifice. The authors concluded that, although such patients inherently carry a higher risk of developing metachronous UTUC, ureteral stenting itself did not appear to further increase this risk. In their cohort, 31 cases of TURBT involved the ureteral orifice. Among these, a ureteral stent was placed in 6 patients, while 25 patients were managed without drainage. After 5–8 years of follow-up, none of the 6 stented patients developed metachronous UTUC, while 4 of the 25 non-stented patients did [21]. On the contrary, Hupe et al. reported different findings in a heterogeneous cohort of 617 patients who underwent TURBT. Their analysis suggested a potential association between ureteral stenting and the development of metachronous UTUC. Among the study population, 78 patients presented with hydronephrosis, 44 of whom were treated with ureteral stenting and 34 with percutaneous nephrostomy. In an additional 35 cases, a ureteral stent was placed during TURBT as a preventive measure rather than for existing hydronephrosis. During a mean follow-up of 15 months, metachronous UTUC occurred in 4 patients who had undergone ureteral stenting, while no cases were observed among those managed with nephrostomy [22].

Still, it was Kiss et al. who fueled the debate about the role of ureteral stenting on metachronous UTUC. In a radical cystectomy cohort, 226 patients presented with hydronephrosis, 53 of whom were managed with a ureteral stent, 61 with percutaneous nephrostomy, and 112 with no drainage. After a mean follow-up of 44 months, 31 (3%) patients presented with metachronous UTUC. After a multivariable regression analysis, the authors concluded that patients undergoing ureteral stenting before radical cystectomy are at higher risk of metachronous UTUC [23]. On the contrary, Miest et al. also analyzed a cohort of 262 radical cystectomy patients with hydronephrosis, of whom 94 underwent ureteral stenting, 36 were treated with nephrostomy, and 132 received no drainage. After a mean follow-up of 55 months, 54 patients (5.1%) developed metachronous UTUC. Their multivariable analysis indicated that preoperative hydronephrosis itself was an independent risk factor for UTUC development, whereas ureteral stenting did not further increase this risk [24]. Similarly, Scilipoti et al., in the first multinational radical cystectomy cohort study, found no evidence that ureteral stenting increases the risk of metachronous UTUC in patients with hydronephrosis, regardless of whether drainage was achieved via stenting or nephrostomy. After a median follow-up of 19 months, outcomes were comparable between the two drainage approaches [25].

In an attempt to reconcile the conflicting evidence and standardize clinical practice, Sountoulides et al. conducted a meta-analysis of the available retrospective studies addressing this issue. The authors concluded that prophylactic stenting after TURBT involving the orifice should be avoided. Indeed, it should be noted that it is standard practice in our department to refrain from stenting when the ureteral orifice is clearly visualized and demonstrates active efflux [26]. On the other hand, Sountoulides et al. suggested that upper urinary tract drainage is mandatory in the presence of hydronephrosis, which can be safely achieved through either ureteral stenting or percutaneous nephrostomy, as both approaches appear to confer a comparable risk for metachronous UTUC [13].

Although concerns about tumor cell seeding during stenting leading to metachronous UTUC have been raised, this issue has never been prospectively assessed. In the absence of such a level of evidence, we evaluated, in a holistic approach, whether ureteral stenting is associated with metachronous UTUC. Nevertheless, it should be highlighted that the findings of the present study should be interpreted within the context of some limitations relevant to its non-blinded nature and to the small number of included participants. Our study was underpowered to exclude a clinically meaningful effect. Based on the previous notion, we could not perform a multivariable time-to-event analysis to assess the impact of specific risk factors and comorbidities, such as tumor characteristics, stent indwelling time, prior intravesical treatment, aging, or smoking, on the rates of developing metachronous UTUC. Similarly, an adjusted approach such as a propensity-based analysis was not feasible due to the limited number of events. Importantly, none of the included patients underwent placement of only a percutaneous nephrostomy. Therefore, comparisons among ureteral stenting, nephrostomy, and no drainage could not be performed. Finally, follow-up was heterogeneous across the cohort, which may have led to underdetection of late metachronous UTUC. Of note, our findings were further mitigated by the fact that we did not assess the impact of ureteral stenting on quality of life. Moreover, even though our results suggest that stenting might not increase the rates of metachronous UTUC, it was beyond the scope of the present study to perform genomic analyses to determine the molecular relationship between the primary bladder tumor and the metachronous UTUC.

## Conclusion

In summary, the present study did not demonstrate an increased risk of metachronous UTUC after stenting during TURBT with urothelial cancer involving the ureteral orifice. Moreover, stenting was not statistically significantly associated with increased recurrences in the urinary bladder, higher cystectomy rates, or worse mortality. Within the limitations of this relatively small heterogeneous observational cohort, no signal of increased UTUC was identified, but clinically relevant harm cannot be excluded, and larger appropriately adjusted studies with longer follow-up remain necessary. Moreover, it should be highlighted that in our everyday clinical practice, we avoid ureteral stenting not only for tumor cell seeding issues but also due to a worse quality of life. Overall, prospective studies with molecular correlation analyses are also warranted to validate our findings and to better define the role of ureteral stenting in this specific clinical scenario.

## Supplementary Information

Below is the link to the electronic supplementary material.

Supplementary Material 1

## Data Availability

The data that support the findings of this study are available from the corresponding author, but restrictions apply to the availability of these data, which were used under licence for the current study and so are not publicly available. The data are, however, available upon request.
