# Supplementary material for: The effect of ureteral stenting on the risk of metachronous upper tract urothelial carcinoma in patients with bladder tumors involving the ureteral orifice
Source: World J Urol. 2026 Oct 5;44(1):725. doi: 10.1007/s00345-026-06839-7 (PMC13638760; doi:10.1007/s00345-026-06839-7)
Supplement: Supplementary file 1 [file 345_2026_6839_MOESM1_ESM.docx]

# Supplementary Table: Indication for Ureteral Stenting (n = 37)

*HN = hydronephrosis; Crea = serum creatinine; LOS = length of hospital stay. "Not specified" indicates delayed stent placement with no complication or obstructive finding documented in the chart.*

## A. Intraoperative stenting during TURBT (n = 27)

| **ID** | **Timing** | **Laterality** | **Indication** | **Preop HN** | **Discharge HN** | **Preop Crea (mg/dl)** | **Discharge Crea (mg/dl)** | **Preop eGFR** | **Discharge eGFR** | **LOS (d)** |
| --- | --- | --- | --- | --- | --- | --- | --- | --- | --- | --- |
| 2 | Intraoperative | Right | Prophylactic | Yes | No | 2.5 | 3.7 | 13 | 16 | 5 |
| 3 | Intraoperative | Left | Prophylactic | Yes | No | 1.8 | 1.7 | 40 | 42 | 5 |
| 12 | Intraoperative | Right | Prophylactic | No | No | 1.5 | 1.2 | 35 | 46 | 4 |
| 32 | Intraoperative | Right | Prophylactic | No | No | 1.3 | 1.3 | 65 | 65 | 4 |
| 45 | Intraoperative | Left | Prophylactic | No | No | 1.0 | 1.2 | 98 | 77 | 14 |
| 47 | Intraoperative | Right | Prophylactic | Yes | No | 1.7 | 1.5 | 44 | 51 | 5 |
| 58 | Intraoperative | Right | Prophylactic | No | No | 1.0 | 1.0 | 99 | 100 | 4 |
| 66 | Intraoperative | Right | Prophylactic | No | No | 1.5 | 1.8 | 48 | 39 | 8 |
| 69 | Intraoperative | Left | Prophylactic | Yes | No | 1.4 | 1.2 | 46 | 56 | 3 |
| 74 | Intraoperative | Right | Prophylactic | Yes | No | 1.2 | 1.2 | 70 | 69 | 21 |
| 96 | Intraoperative | Right | Prophylactic | Yes | No | 1.1 | 1.1 | 55 | 56 | 8 |
| 109 | Intraoperative | Right | Prophylactic | Yes | No | 1.1 | 0.8 | 52 | 75 | 25 |
| 117 | Intraoperative | Right | Prophylactic | No | No | 1.1 | 1.1 | 61 | 65 | 5 |
| 134 | Intraoperative | Left | Prophylactic | Yes | No | 1.7 | 1.2 | 38 | 54 | 4 |
| 153 | Intraoperative | Right | Prophylactic | No | No | 0.7 | 1.1 | 106 | 73 | 11 |
| 163 | Intraoperative | Left | Prophylactic | Yes | No | 1.4 | 1.1 | 34 | 44 | 5 |
| 177 | Intraoperative | Right | Prophylactic | Yes | No | 1.6 | 1.6 | 46 | 46 | 5 |
| 179 | Intraoperative | Left | Prophylactic | Yes | No | 1.3 | 1.2 | 52 | 57 | 4 |
| 185 | Intraoperative | Right | Prophylactic | No | No | 1.0 | 1.1 | 71 | 64 | 6 |
| 191 | Intraoperative | Bilateral | Prophylactic | Yes | No | 0.9 | 2.0 | 66 | 25 | 5 |
| 194 | Intraoperative | Right | Prophylactic | Yes | No | 1.4 | 1.3 | 51 | 46 | 3 |
| 199 | Intraoperative | Left | Prophylactic | Yes | No | 1.3 | 1.4 | 51 | 47 | 5 |
| 206 | Intraoperative | Bilateral | Prophylactic | Yes | No | 1.0 | 1.2 | 59 | 48 | 5 |
| 211 | Intraoperative | Left | Prophylactic | Yes | Yes | 2.2 | 1.8 | 31 | 39 | 4 |
| 232 | Intraoperative | Left | Prophylactic | No | No | 1.3 | 1.3 | 40 | 40 | 4 |
| 234 | Intraoperative | Bilateral | Prophylactic | Yes | No | 2.2 | 2.2 | 29 | 29 | 5 |
| 236 | Intraoperative | Right | Prophylactic | No | No | 0.8 | 0.8 | 89 | 89 | 4 |

## B. Delayed stenting, within 4 weeks after TURBT (n = 10)

| **ID** | **Timing** | **Laterality** | **Indication** | **Preop HN** | **Discharge HN** | **Preop Crea (mg/dl)** | **Discharge Crea (mg/dl)** | **Preop eGFR** | **Discharge eGFR** | **LOS (d)** |
| --- | --- | --- | --- | --- | --- | --- | --- | --- | --- | --- |
| 21 | Delayed | Right | Therapeutic, Rising creatinine | No | No | 1.1 | 0.9 | 100 | 107 | 5 |
| 31 | Delayed | Left | Therapeutic, Rising creatinine | No | No | 0.8 | 0.8 | 96 | 96 | 4 |
| 41 | Delayed | Left | Therapeutic, Rising creatinine | No | No | 1.2 | 1.0 | >60 | >60 | 6 |
| 146 | Delayed | Left | Therapeutic, Rising creatinine | No | No | 0.8 | 1.0 | 97 | 87 | 4 |
| 150 | Delayed | Right | Therapeutic, Rising creatinine | No | No | 0.7 | 0.6 | 89 | 90 | 5 |
| 166 | Delayed | Left | Therapeutic, Rising creatinine | Yes | No | 0.8 | 0.9 | 86 | 83 | 5 |
| 168 | Delayed | Right | Therapeutic, Rising creatinine | No | No | 0.7 | 0.7 | 97 | 96 | 3 |
| 181 | Delayed | Right | Therapeutic, Rising creatinine | No | No | 2.9 | 5.1 | 19 | <10 | 15 |
| 233 | Delayed | Right | Therapeutic, Rising creatinine | No | No | 1.0 | 0.9 | 77 | 88 | 7 |
| 244 | Delayed | Right | Therapeutic, Rising creatinine | No | No | 1.1 | 1.5 | 66 | 46 | 5 |
